# Protective Role of Oxides on Pb-Free Halide Perovskite Surfaces: Interfacial Effects and Excitonic Optical Properties from First-Principles

**DOI:** 10.1021/acsami.6c00873

**Published:** 2026-06-08

**Authors:** Maurizia Palummo, Costanza Borghesi, Azusa Muraoka, Suraya Shaban, Shuzi Hayase, Daniele Varsano, Koichi Yamashita, Giacomo Giorgi

**Affiliations:** † Department of Physics & INFN, 540549 Universitá di Roma “Tor Vergata,” Via della Ricerca Scientifica 1, Roma 00133, Italy; ‡ Centro S3, 720961 CNR-Istituto Nanoscienze, Via G. Campi 213/a, Modena 41125, Italy; § Department of Civil & Environmental Engineering (DICA), 9309 The University of Perugia, Via G. Duranti 93, Perugia 06125, Italy; ∥ CIRIAF - Interuniversity Research Centre, University of Perugia, Via G. Duranti 93, Perugia 06125, Italy; ⊥ Graduate School of Science, Japan Women’s University, Tokyo 112-8681, Japan; # 13133 i-PERC, the University of Electro-Communications, 1-5-1 Chofugaoka, Chofu, Tokyo 182-8585, Japan; ∇ Graduate School of Nanobioscience, Yokohama City University, Yokohama 236-0027, Japan; ○ CNR-SCITEC, Perugia 06123, Italy

**Keywords:** photoconversion, optoelectronic properties, solar energy, crystalline interface models

## Abstract

In this work, the impact of a GeO_2_ overlayer on the stability and optoelectronic properties of Pb-free Cs­(Sn_1–_ *_x_* Ge_*x*_)­I_3_ perovskites is systematically investigated using first-principles calculations. Ge incorporation is found to substantially reduce the exciton binding energy relative to pristine CsSnI_3_, thereby promoting more efficient electron–hole separation and enhancing the potential for carrier extraction. Guided by this trend, Cs­(Sn_1–*x*_Ge_*x*_)­I_3_ (*x* = 0.25) slab models exposing the stable (001) surface were constructed, and both CsI- and MI_2_-terminated facets (M = Sn, Ge, as well as Ge-only) were examined. The resulting perovskite/GeO_2_ heterointerfaces display a pronounced dependence of structural rearrangements and band edge alignment on both composition and termination, allowing the identification of specific configurations that best preserve the desirable optoelectronic response. To better mirror experimental conditions, crystalline interface models were complemented by amorphous GeO_2_ structures, thus capturing the structural complexity of realistic germania capping layers. Taken together, these results provide a microscopic picture of how GeO_2_ overlayers can stabilize (Sn,Ge)-based halide perovskites while retaining electronic characteristics compatible with high-efficiency photovoltaic operation.

## Graphical abstract

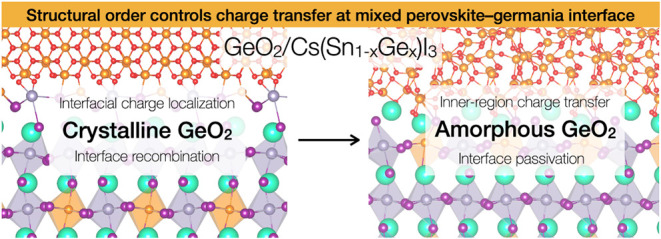

## Introduction

Hybrid organic–inorganic halide perovskites (OIHPs) represent the major breakthrough in solar energy conversion over the last decades.^1^ From the very initial work of Miyasaka et al.,^2^ where the suitability of 3D bulk OIHPs in photoconversion has been demonstrated, new unprecedented photoconversion efficiencies (PCEs) have been reported, which nowadays reach and pass 26%.^3^ Such class of compounds, characterized by the stoichiometric relationship ABX_3_ (A = short-chain organic cation, usually CH_3_NH_3_^+^ and HC­(NH_2_)_2_^+^; B = Pb^2+^, Sn^2+^, and less commonly Ge^2+^; X = halides) embodies a series of features^4–7^ that confer unique photoconversion properties, such as long carrier diffusion and lifetime, and not secondarily, an extremely low cost of processability.^8^ Several papers and reviews have been published since 2009 aiming at characterizing—both experimentally and theoretically—such extraordinary class of compounds (see, e.g., among the many refs 9,10 ). Many studies have been devoted to address the two intertwined issues that continue to hinder the large-scale exploitation of 3D organic–inorganic halide perovskites (OIHPs) in commercial devices, i.e., (i) the presence of a toxic element in the B-site of the compounds and (ii) the inherent instability of the 3D bulk class of compounds toward heat and moisture. The latter is enhanced by the volatility due to the presence of short-chain, *highly hydrophilic* organic moieties in the A-site. To mitigate this instability, a dimensionality-reduction strategy, from 3D to mixed 3D/2D or fully 2D systems, has been thus suggested.^11–19^ Although this approach generally implies a modest reduction in power conversion efficiency (PCE), state-of-the-art values remain remarkably high, reaching 22% in alternating cations in the interlayer space (ACI) architectures^20^ and up to 24.35% in the Ruddlesden–Popper motif.^21^ This strategy consists on replacing short-chain organic cations with longer, *highly hydrophobic* aliphatic (e.g., butylammonium, BA = CH_3_(CH_2_)_3_NH_3_^+^) or aromatic (e.g., phenethylammonium, PEA = C_6_H_5_(CH_2_)_2_NH_3_^+^) cations, which significantly enhance the structural stability of the resulting mixed-dimensional systems.

Beyond structural concerns, the environmental challenge of lead remains critical and probably the most tedious to solve. Efforts to address this issue have primarily focused on two strategies: homovalent replacement of Pb with Sn (either fully or partially—see, e.g., among the others, refs 22,23 ), and heterovalent substitution of Pb pairs with two metallic centers with +1/+3 oxidation state.^24–29^ Both processes suffer from side effects that potentially limit their applications. Sn at the B-site oxidizes to the +4 state more readily than Pb, accelerating the degradation of the perovskite solar cell (PSC). On the other hand, two different metals that occupy 50% of the B-site can decrease the dispersion of the band edge,^30–32^ inducing reduced performance of the final PSC. However, attention has turned to mixed +2 oxidation state B-site halide perovskites (HPs),^33^ and in particular to Sn-based ones. Indeed, forming solid solutions with Ge has been reported to improve the final alloyed system, compared to pristine Sn-based. While, indeed, pure germanium HPs^34^ face intrinsic limitations as solar harvester, stemming from a marked lone-pair effect and for their bandgap slightly larger than that of Pb and Sn counterparts,^35^ on the other hand, introducing Ge into Sn-based ones has been demonstrated to confer stability and improve the overall optoelectronic features. In this scenario, Hayase et al., mixing cations in both the A-site (FA, MA) and the B-site (Sn,Ge), proposed at first a (Sn,Ge)-mixed series of HPs whose bandgaps, measured by photoacoustic spectroscopy, fall within the optimal range for single-junction solar devices (1.40–1.53 eV). This finding supports the applicability of such mixed systems in solar cells: as reported, ∼5% doping Ge into the perovskite improves the efficiency up to 4.48% without encapsulation and, interestingly, upon doping the (Sn,Ge)-mixed HPs retain 80% of the original performance.^36^ Still Hayase et al.^37^ studied the role GeI_2_ in (Sn,Ge)-mixed PSCs reporting an overall reduced device trap densities (10^15^–10^17^ cm^–3^ when no Ge is added to 10^8^–10^14^ cm^–3^ when 5% of Ge is added), long-lived carrier lifetime (τ = 5.04 ns), higher mobility (98.27 cm^2^V^–1^s^–1^), longer diffusion length (>1 μm), and a final PCE of 7.9%. Here, authors ascribe the overall improvements in such mixed systems to the ability of Ge atoms to passivate the trap states. The impact of the different transparent conductive oxide substrates on the (Sn,Ge)-mixed PSCs has been investigated by Hamada et al.,^38^ along with a comparison between inverted and standard structure: exploiting fluorine-doped tin oxide as a substrate led to a noticeable increase of the final efficiency of the PSC from 7.72% to 9.24%.

On the theoretical side, Zhou et al.^39^ by means of a combination of standard DFT calculations both exploiting pure and hybrid functionals, report a complete description on the defect formation in such (Sn,Ge)-alloyed HPs. In detail, for different concentrations of Ge in Cs­(Ge_1–*x*_Sn_*x*_)­I_3_, they found that the formation of antisites (I_Sn_ and I_Ge_) may have a beneficial impact on the final properties of the *x* = 0.5 system due to the combined effect of alloying on both band edges and defect states. Focusing on the role played by the native oxide (GeO_2_) formed at the surface of CsSn_0.5_Ge_0.5_I_3_ mixed perovskite, Chen et al.^40^ have demonstrated the benign effects of such formation on the overall working principle of the final device: the utilization of these perovskites is primarily driven by their exceptional stability, which stems from the spontaneous formation of a robust native germania layer. This ultrathin passivation layer effectively encapsulates and protects the perovskite surfaces, mitigating degradation pathways. Still, the authors demonstrate that alloying with Ge­(II) in CsSnI_3_ markedly enhances both stability and air tolerance. The rapid formation of a uniform native oxide surface layer on the mixed perovskite provides superior passivation, outperforming even the benchmark MAPbI_3_ perovskite under ambient conditions. As a result, CsSn_0.5_Ge_0.5_I_3_ solid-solution HPs may be exploited in PSCs reaching a PCE of 7.11% and are stable upon continuous operation under 1-sun illumination for over 500 h. Despite the growing interest in (Sn,Ge)-alloyed perovskites due to their appealing properties, our understanding of their chemistry remains limited. Even less is known about the mechanisms underlying their remarkable stability, particularly the surprising passivation effect provided by native oxide formation.^40,41^ Accordingly, we consider it mandatory to further shed light on these systems paying particular attention to the properties of such (Sn,Ge)-alloyed HP surfaces, in their reactivity toward oxidizing agents and, last but not least, to the interfaces formed with native oxides, i.e., GeO_2_.^40^ Clearly, our focus is not the growth of oxide, which we cannot theoretically mimic by means of standard DFT calculations: in view of their potential implementation in PV, we study and theoretically characterize the structural and electronic features of the final coupled perovskite/germania systems. To the best of our knowledge, this work is one of the first to focus on the theoretical study of the interface between halide perovskites and oxide compounds.

## Computational Details

Ground-state density functional theory (DFT) calculations were performed using the Vienna ab initio simulation package (VASP).^42–45^ The Generalized Gradient Approximation (GGA), as parametrized by Perdew, Burke, and Ernzerhof (PBE),^46^ was used to calculate the exchange-correlation (XC) energy. The projector augmented wave (PAW) potentials^47,48^ were similarly used along with an energy cutoff of 600 eV. The semiempirical DFT-D3 corrections with Becke–Johnson damping^49,50^ were included to account for van der Waals interactions. The Brillouin Zone (BZ) of the different systems was sampled with Γ-centered grids of different mesh, according to the size of the system: in particular, an 8 × 8 × 6 grid was used for optimizing the bulk orthorhombic γ-CsSnI_3_ pristine cell and Cs­(Sn_1–*x*_Ge_*x*_)­I_3_ (*x* = 0.25, 0.50) alloyed systems, while a 12 × 12 × 16 one was used for rutile GeO_2_ (hereafter *r*-GeO_2_, (P4_2_/*mnm*)). The atomic positions and bulk lattice parameters of all the materials considered (CsSnI_3_, CsGeI_3_, Cs­(Sn_1–*x*_Ge_*x*_)­I_3_ (*x* = 0.25, 0.50), and GeO_2_) were relaxed until all forces were lower than 0.005 eV/Å, while for larger systems (surfaces and interfaces) the threshold was set to 0.05 eV/Å. Preliminary analysis on the polymorph stability (see Figure 1 and Table 1) confirms the experimental trend with the orthorhombic system as the most stable at room temperature, followed by the tetragonal, and the cubic.^51,52^ This ordering has also been reported at the theoretical level,^53^ further validating our setup. Following such results, we considered the orthorhombic polymorph for further surface/interface property analysis.^52^

**Figure 1:**
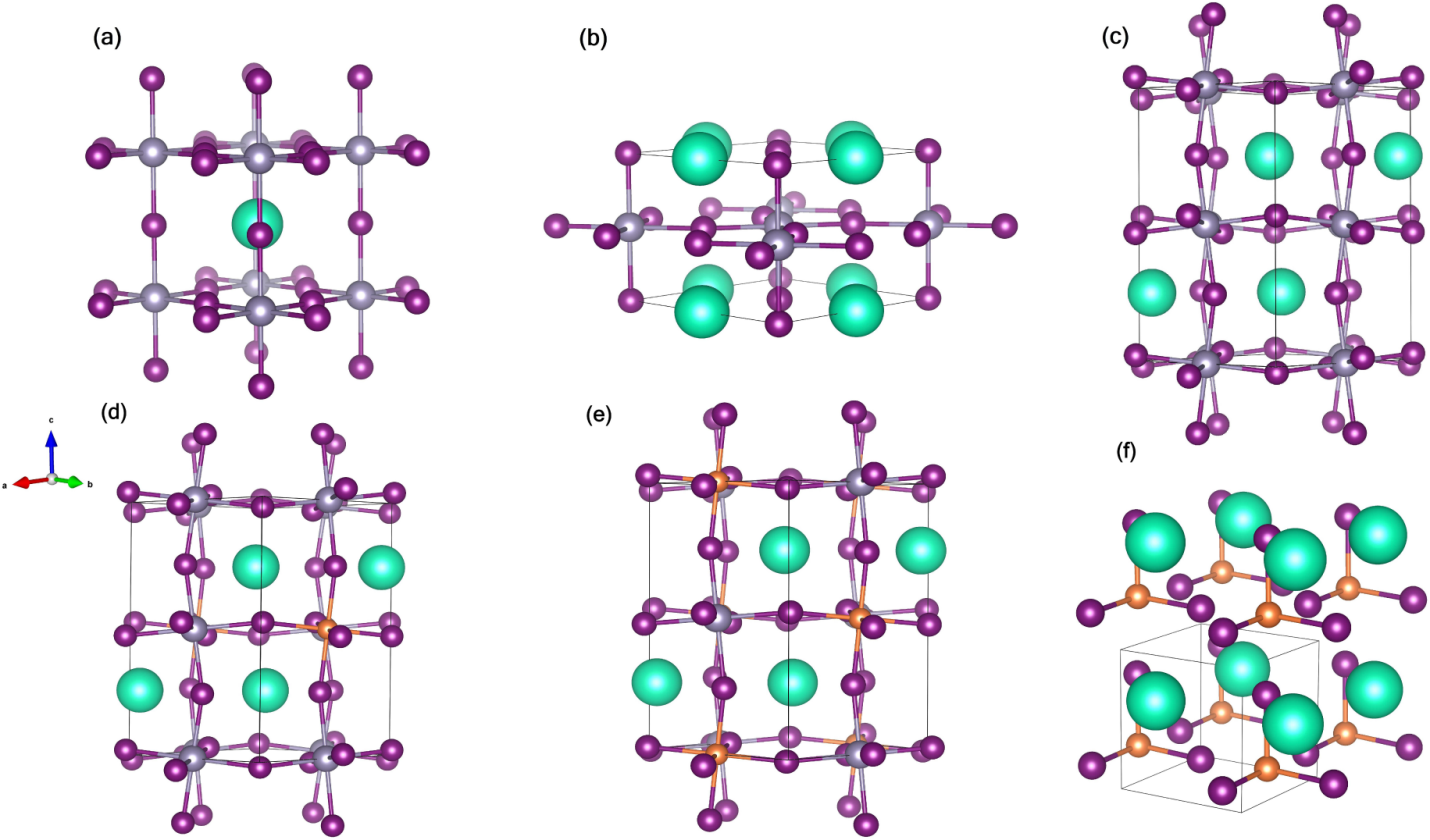
PAW/PBE-optimized atomic structure of (a) cubic, α- (b) tetragonal, β, (c) orthorhombic, γ-CsSnI_3_, (d) Cs­(Sn_1–*x*_Ge*_x_*)­I_3_ (*x* = 0.25), (e) Cs­(Sn_1–*x*_Ge*_x_*)­I_3_ (*x* = 0.50) alloyed systems, (f) (2 × 2 × 2) supercell of CsGeI_3_ [Mauve: Sn; Orange: Ge; Purple: I; Cyan: Cs atoms].

**Table 1:** PAW/PBE Calculated Structural Parameters (Lattice Constants and Volume), Energy Difference (Δ*E*) Relative to the Most Stable Polymorph, Formation Energy (*E*_form_, eV) of the Cs­(Sn_1–*x*_Ge_*x*_)­I_3_ Intermediate Alloys, and Energy Bandgap (*E*_gap_, eV) of the Systems of Interest^a^

CsSnI_3_ Polymorph				Cs(Sn_1–*x*_Ge_*x*_)I_3_ Alloys		CsGeI_3_
	Cubic (α, *Pm3m*, *Z* = 1)	Tetragonal (β, P_4/*mbm*_, *Z* = 2)	Orthorhombic (γ, *Pnma*, *Z* = 4)	*x* = 0.25	*x* = 0.50	Trigonal (*R*3*m* *Z* = 1)
Lattice param. (Å)	*a* = 6.17 (*a* = 6.22)^52^ (*a* = 6.13)^75^	*a* = 8.61; *c* = 6.26 (*a* = 8.77; *c* = 6.26)^52^ (*a* = 8.61; *c* = 6.11)^75^	*a* = 8.82; *b* = 8.39; *c* = 12.36 (*a* = 8.69; *b* = 8.64; *c* = 12.38)^52^ (*a* = 8.59; *b* = 8.54; *c* = 12.24)^75^	*a* = 8.66; *b* = 8.44; *c* = 12.09	*a* = 8.52; *b* = 8.43; *c* = 12.09	*a* = 5.93 (*a* = 5.98)^76^ (*a* = 5.98)^34^
Vol. (Å^3^)	234.54 (240.52,^52^ 230.39 ^75^)	463.77 (481.77,^52^ 453.03 ^75^)	915.27 (929.47,^52^ 897.84 ^75^)	892.64	869.08	208.55 (213.98)^34,76^
Δ*E* (eV/f.u.)	+0.065	+0.02	0.0			
*E*_form_ (eV)				0.07	0.059	
*E*_gap_ (eV)			0.70	0.623	0.612	0.680

a Data in parentheses are from experiments (*Z*, formula unit per cell).

To reveal the role of electron–hole interactions and local-field effects, the optical spectra were calculated using the Yambo code^54,55^ based on preliminary self-consistent and nonself-consistent DFT calculations performed with the Quantum ESPRESSO suite.^56,57^ Fully relativistic norm-conserving pseudopotentials (including spin–orbit coupling, SOC)^58^ were employed, together with a kinetic-energy cutoff of 70 Ry and a shifted 6 × 6 × 6 *k*-point grid to converge the ground-state charge density. All calculations were performed using the PBE XC functional consistently with the previous calculations performed with VASP. Several studies have shown that an accurate description of the quasi-particle band structure of similar halide perovskites is highly sensitive to the underlying XC description and to nonharmonic lattice effects.^59–62^ To avoid the well-known ambiguities related to the choice of the starting point in GW calculations of lead-free perovskites^63,64^ considering the high-computational cost and, given the impact of anharmonicity^60,61^ —we opted here not to perform GW simulations but to apply rigid scissor operators to compensate the PBE + SOC gap underestimation and bring the first optical peak positions in agreement with the onset of the available experimental absorption data.^40,65–66^ The scissor operator values turn out to be 1.25 eV for tin-based and 0.9 eV for germanium and the alloyed perovskite. Optical spectra were obtained by solving the Bethe-Salpeter Equation (BSE) considering an 8 × 8 × 6 (8 × 8 × 8 for the Ge-case) Γ centered *k*-grid and four valence and conduction states. Furthermore, we adopted the Tamm–Dancoff approximation, and the double-grid technique, as implemented in the Yambo code,^67^ has been employed to speed up the convergence of the optical spectra with respect to the *k*-points grid.

Concerning surface termination, we selected the (001) orientation since, together with the (110) surface, it is reported to have lower cleavage energy in γ-CsSnI_3_. This implies that the (001) surface is preferentially exposed and more prone to the formation of heterostructures with protection layers.^68^ Accordingly, the (001) surface is selected in the following calculations to investigate the interfaces formed with GeO_2_, both in its crystalline (*r*-GeO_2_) and amorphous structure. Not secondarily, previous theoretical simulations focused on the same surface^53^ providing data for comparison. GeO_2_ is well known to exist both as quartz and rutile. While the former α-GeO_2_ is metastable at ambient conditions,^69^ the rutile polymorph represents the naturally occurring GeO_2_ form.^70–72^ Accordingly, in order to study Cs­(Sn_1–*x*_Ge_*x*_)­I_3_–GeO_2_ interfaces we focused our attention on rutile. Paying attention to the *r*-GeO_2_ slab, the construction of such interfaces may be based on two different approaches. The first and the most common consists in keeping fixed the lateral parameters of the whole interface to those of the substrate. Within this framework, we found the (100) surface to provide the best lattice matching with the lateral parameter of Cs­(Sn_1–*x*_Ge_*x*_)­I_3_ (*x* = 0.25). While such approach may better mimic the experimental growth conditions—and has been successfully applied in our previous theoretical investigations of several interfacial systems^73,74^ at the same time tends to (over)­localize the interfacial strain predominantly on a single component (in this case, GeO_2_). This effect is further exacerbated when the simulation cell is minimized to limit computational cost. Consequently, we chose to share the total stress equally between the two components, thus applying tensile (compressive) stress on the Cs­(Sn_1–*x*_Ge_*x*_)­I_3_ (*r*-GeO_2_) component. Within this scheme, the best lattice match was obtained for the Cs­(Sn_1–*x*_Ge_*x*_)­I_3_ (001) surface interfaced with the (001) surface of *r*-GeO_2_. Further discussion of the interface assembly procedure is reported below. We first considered a 6-layer-thick rutile GeO_2_ bulk consisting in 48 GeO_2_ units to construct the initial crystalline interface. Following the 0 K optimization, to better mimic the experimental findings, we have assembled further interfaces with amorphous germania. To this end, we have expanded the germania volume (by ∼10%) breaking the symmetry of the initially optimized structure of the oxide supercell. A series of *ab initio* molecular dynamics (AIMD) simulations in the NVT canonical ensemble was then performed. The system was first thermalized at 4500 K for ∼25 ps (τ = 1.0 fs), and then quenched toward room temperature. The quenching procedure involved an initial relatively slow cooling rate (2.0 × 10^14^ K s^–1^) followed, below 2500 K, by an increased cooling rate of 4.5 × 10^14^ K s^–1^. The system obtained in this way was finally reoptimized at the DFT level. In all the interfaces here considered, a sufficient amount of vacuum is added along the nonperiodic direction in order to avoid any possible spurious interaction between replicas.

## Results and Discussion

### Structural, Electronic, and Optical Properties of Perovskites

We initially calculated and reported in Table 1 the optimized lattice parameters of the three CsSnI_3_ polymorphs, shown in Figure 1a–c along with the energies (Δ*E*) relative to the most stable CsSnI_3_ polymorph, values that are in good agreement with previously reported theoretical^75^ and experimental data.^51,52^ In detail, the bulk orthorhombic CsSnI_3_ structure is characterized by an Sn–I bond length of 3.13 Å, both along the apical and equatorial directions, with corresponding Sn–I–Sn bond angles of 160° and 152.2°, respectively. To complete the scenario, we also report the optimized structure CsGeI_3_ (see Figure 1f), its structural parameters (still in Table 1) and the optimized structures of Cs­(Sn_1–_ *_x_* Ge_*x*_)­I_3_ (*x* = 0.25, 0.50) alloyed systems (see Figure 1d,e). Interestingly, we observe the experimentally reported pattern of two alternating Ge–I bond lengths, short (2.81 Å) and long (3.14 Å), a feature that will be particularly recursive also in the interfacial systems.

To create the (Sn,Ge)-alloyed systems, we first randomly substituted one Sn atom with a Ge atom in the bulk orthorhombic γ-CsSnI_3_ unit cell (see Figure 1c) thereby obtaining Cs­(Sn_1–*x*_Ge_*x*_)­I_3_ (*x* = 0.25, Figure 1d). This substitution induces an overall shrinkage of the volume of ∼2.3% (from 914.64 Å^3^ in the pristine cell to 893.14 Å^3^ in the final *x* = 0.25 Ge-alloyed one). Furthermore, as additional confirmation of the validity of our approach, we calculated the bandgap for γ-CsSnI_3_, obtaining a value (@PBE/PAW level of theory) of 0.70 eV. Replacing 25% Sn with Ge, we obtain a modest but observable narrowing of the gap (0.63 eV), confirming the results of Chang et al.,^77^ who report a progressive decrease of the alloy bandgap for 0 < *x* < 0.50 and a symmetrical increase in the range 0.5 < *x* < 1.0. The same trend is also observed when relativistic effects are included in the calculations. The electronic band structure calculated at the PBE + SOC level of approximation for the two pristine CsSnI_3_ and CsGeI_3_ systems along with that for the Cs­(Sn_1–*x*_Ge_*x*_)­I_3_ (*x* = 0.50) alloy is reported in Figure S1 in the Supporting Information section. The corresponding PDOS, which are important for rationalizing the different roles of excitonic effects in the optical spectra, are reported in Figure 2.

**Figure 2:**
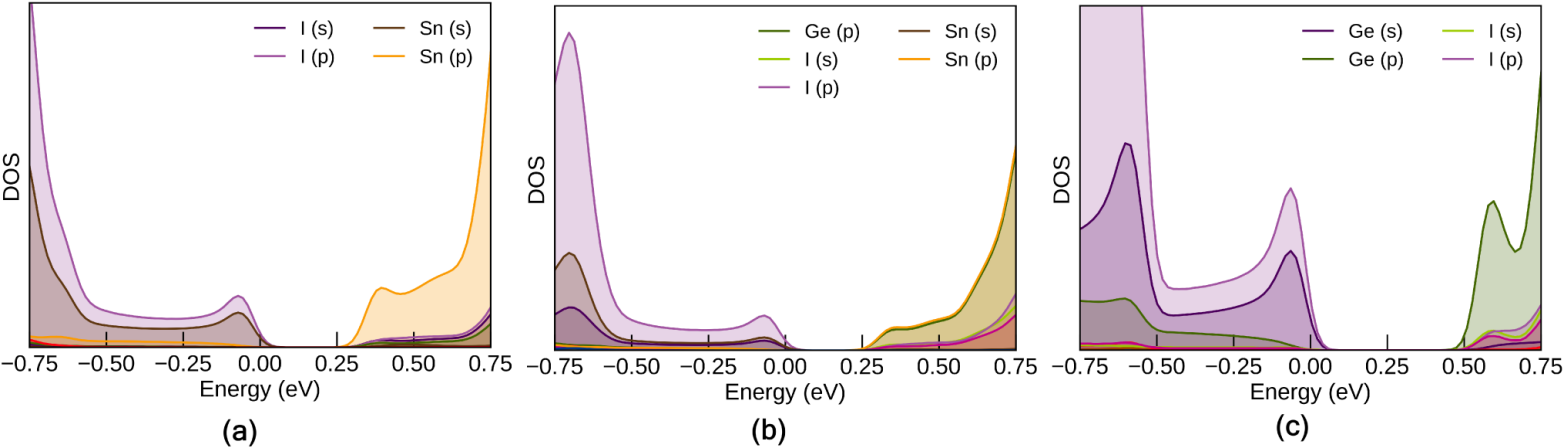
PAW/PBE + SOC-calculated Projected Density of States of (a) γ-CsSnI_3_, (b) Cs­(Sn_1–*x*_Ge*_x_*)­I_3_ (*x* = 0.50) alloyed system, and (c) CsGeI_3_.

To evaluate the relevance of the mixed alloy with respect to the pristine compounds, we computed and compared the optical spectra of CsSnI_3_, CsSn_0.5_Ge_0.5_I_3_, and CsGeI_3_ (see Figure 3). The spectra were calculated both including (red solid lines) and neglecting (blue solid lines) excitonic and local-field effects. Our results show that the red shift and the enhancement of the main absorption peaks induced by electron–hole interactions are significant only in the Sn-based perovskite. In contrast, excitonic effects are much weaker in the Ge-containing compound and in the mixed alloy, whose spectra remain very close to the independent-particle ones. Due to the large dimensions of the BSE matrix, we employed the Haydock solver to obtain absorption spectra, though this approach precludes direct access to excitonic eigenvalues. Therefore, the exciton binding energies were estimated from the difference between the absorption onset calculated with and without electron–hole interactions (red and blue curves in Figure 3). This estimate yields a binding energy of approximately 100 meV for CsSnI_3_, whereas values at least 1 order of magnitude smaller are obtained for the other two compounds. Therefore, the exciton binding energies were estimated from the difference between the absorption onset calculated with and without electron–hole interactions (red and blue curves in Figure 3). In this regard, it is important to emphasize that neither polaronic effects nor the ionic contribution to dielectric screening were included in the present work. As reported in the literature for some halide perovskites,^59,63,78^ the inclusion of these effects is expected to enhance the overall screening and consequently reduce the calculated exciton binding energies, likely alleviating the discrepancy with ref 59. At the same time, these corrections are not expected to modify the qualitative trends identified in the present study, since our analysis considers only the electronic contribution to dielectric screening, treated consistently across all the systems investigated. The analysis of the projected density of states (PDOS, Figure 2) in the vicinity of the VBM and CBM provides a clear explanation of this behavior. In CsSnI_3_, the states near the VBM (CBM) are dominated by the *s* (*p*) orbitals of Sn, which are highly hybridized with I *p* ones, whereas in (Sn,Ge)-mixed and Ge-pure systems, the VBM progressively acquires a dominant contribution from the *p* orbitals of I, while the CBM is primarily localized on the *p* orbitals of Ge (of both Ge and Sn in the (Sn,Ge)-mixed structure). This gradual change from an intra-atomic excitation character, dominated by Sn­(*s*) → Sn­(*p*) transition in CsSnI_3_ to a predominantly interatomic character, namely I­(*p*) → Sn­(*p*), Ge­(*p*) transitions in the Ge-based and alloyed systems explains the observed reduction of excitonic effects. In such cases, the electron–hole interaction becomes less effective when the electron and hole are more spatially separated or reside on different atomic species.

**Figure 3:**
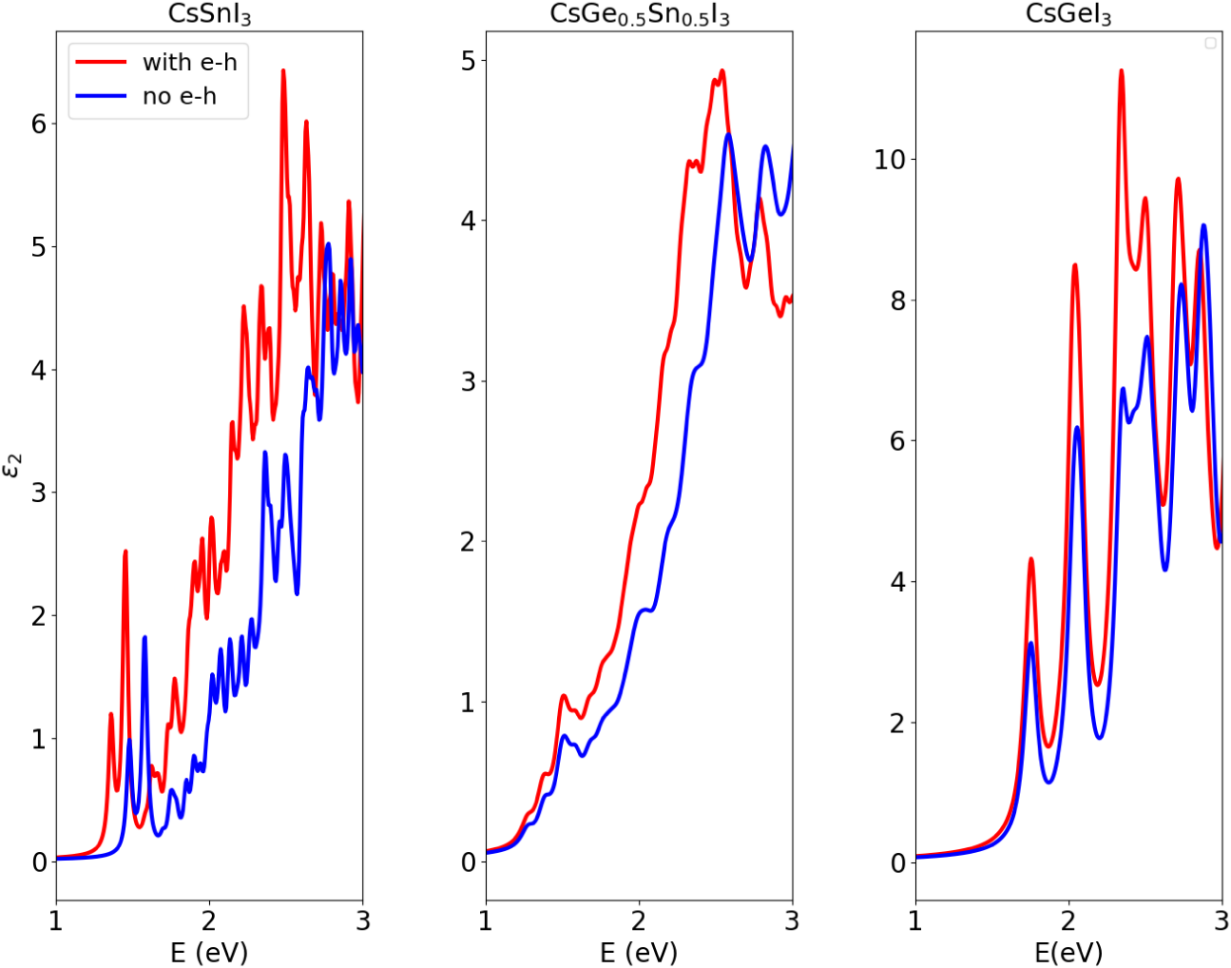
Optical spectra of the three different parent compounds, (left) orthorhombic γ-CsSnI_3_, (middle) CsSn_0.5_Ge_0.5_I_3_, and (right) rhombohedral CsGeI_3_, calculated with and without electron–hole interaction.

### From Bulk to Surface: Slab Thickness Convergence

Passing to the surface analysis, we check at first the slab thickness convergence of symmetric slabs vs the formation energy on the pristine γ-CsSnI_3_ (001) surface, paying initial attention to the MI_2_-terminated one. To take into account the nonstoichiometric nature of the slab we have to include another contribution that takes into account the excess of SnI_2_ (which anyway is constant) following the equation:

1 $$E_{form}=\frac{E_{slab}\left(11s\right)-(n\times \mu_{CsSnI_{3}}+2\times \mu_{SnI_{2}})}{2A}$$

Here, *s* in *E*_slab_(11*s*) is the number of bulk units along (001) direction with relative energy (*E*_slab_). Each bulk unit contains two layers of perovskite along the (001) direction and *s* tested values are 2, 3, and 4, respectively (see Figure 1c). $\mu_{{\mathrm{CsSnI}}_{3}}$ (and relative amount, *n*) and $\mu_{{\mathrm{SnI}}_{2}}$ are the chemical potentials for CsSnI_3_ (γ-CsSnI_3_), and SnI_2_ (space group 12, *C*2/*m*),^79^ respectively. *n* values considered here are accordingly 8, 12, and 16, respectively. A is the in-plane surface area. After calculating *E*_form_ for *s* = 2, 3, 4 we observed a plateau (*E*_form_= 0.26 J m^–2^, *s* = 2; *E*_form_ = 0.28 J m^–2^, *s* = 3; and *E*_form_ = 0.29 J m^–2^, *s* = 4) and accordingly considered sufficient 3 units (6 layers) thick slabs for all the calculations, for both pristine γ-CsSnI_3_ and γ-Cs­(Sn,Ge)­I_3_ (001) surfaces. The slab thickness convergence test is followed by the calculation of the formation energy of the different terminations of the Cs­(Sn_1–*x*_Ge_*x*_)­I_3_ (*x* = 0.25) (001)-oriented slabs. In particular, we observe how both terminations, namely CsI and MI_2_ (M = Sn, Ge), prefer germanium to the outermost layer (in the former case, we mean the layer immediately below the CsI layer at the very surface). In particular, by looking at Figure 4, where all the optimized structures of the perovskite slabs are reported, for the MI_2_ termination, the formation energy of the structure depicted in Figure 4b (M_2_ = Ge only) is lower by 0.115 eV than that in Figure 4a (M_1_ = Sn,Ge). For the CsI termination , the slab with a purely Ge layer immediately beneath the surface (M_2_) is more stable than that containing a mixed (Sn,Ge)­I_2_ layer (still immediately beneath the surface) (M_1_, Figure 4c) by 0.124 eV. This indicates a general tendency of Ge atoms to segregate toward the surface. In the following paragraph we describe the construction of the interface between each perovskite slab and germania, without further analyzing the relative thermodynamic stability of the slab models introduced above.

**Figure 4:**
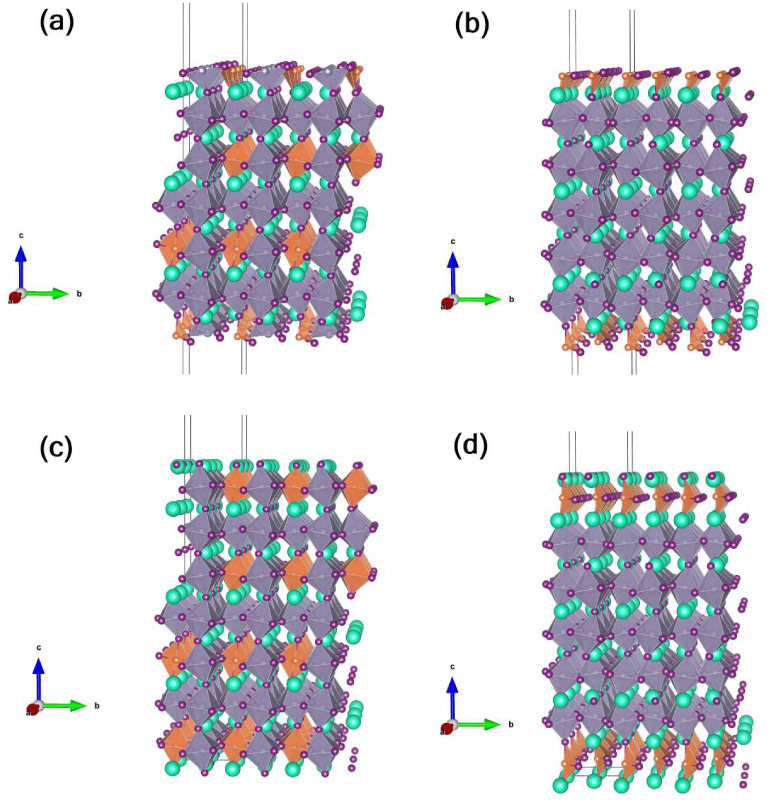
PAW/PBE-optimized structure of (a–b)­MI_2_-terminated and (c–d) CsI-terminated (001)-oriented slabs of γ-Cs­(Sn_1-x_Ge_x_)­I_3_ (*x*=0.25). M = Ge only at the surface (GeI_2_) in (b) and at the subsurface (CsI termination) in (d). Here, for the sake of visualization polyhedral representation is reported. [Mauve: Sn; Orange: Ge; Purple: I; Cyan: Cs atoms].

### Interface Formation with GeO_2_

We have successfully assembled and analyzed the interfaces formed between (1 × 1) Cs­(Sn_1–*x*_Ge_*x*_)­I_3_ (001)-oriented surfaces and a (2 × 2) supercell of *r*-GeO_2_, still (001)-oriented. When studying any interface, the primary consideration is the stress arising from the lattice mismatch between its components, as detailed in the Computational Details section. If we were to keep the lattice parameters of Cs­(Sn_1–*x*_Ge_*x*_)­I_3_ fixed, the germania slab would experience extreme stress. This is because the PAW/PBE-optimized lattice parameters for its (2 × 2) supercell are *a* = *b* = 8.88 Å. To mitigate this issue, we applied a tensile stress to the bottom component and a compressive stress to the top component of the interface. This approach ensures that both Cs­(Sn_1–*x*_Ge_*x*_)­I_3_ and *r*-GeO_2_ experience a stress of ∼3.6% relative to their fully optimized counterparts. This level of stress is reasonable, as demonstrated in Figure 5, which compares the electronic characteristics of stressed versus relaxed structures for the two bulk systems. Notably, the electronic features (Density of States, DOS, and band structure/dispersion) are largely preserved also under these stressed conditions. Therefore, the lateral parameters used for all interfaces are *a* = 8.765 Å and *b* = 8.665 Å. Table 2 presents the main electronic features of the halide perovskite slabs involved in the interface formation. The four optimized interface structures formed with *r*-GeO_2_ are shown in Figure 6.

**Figure 5:**
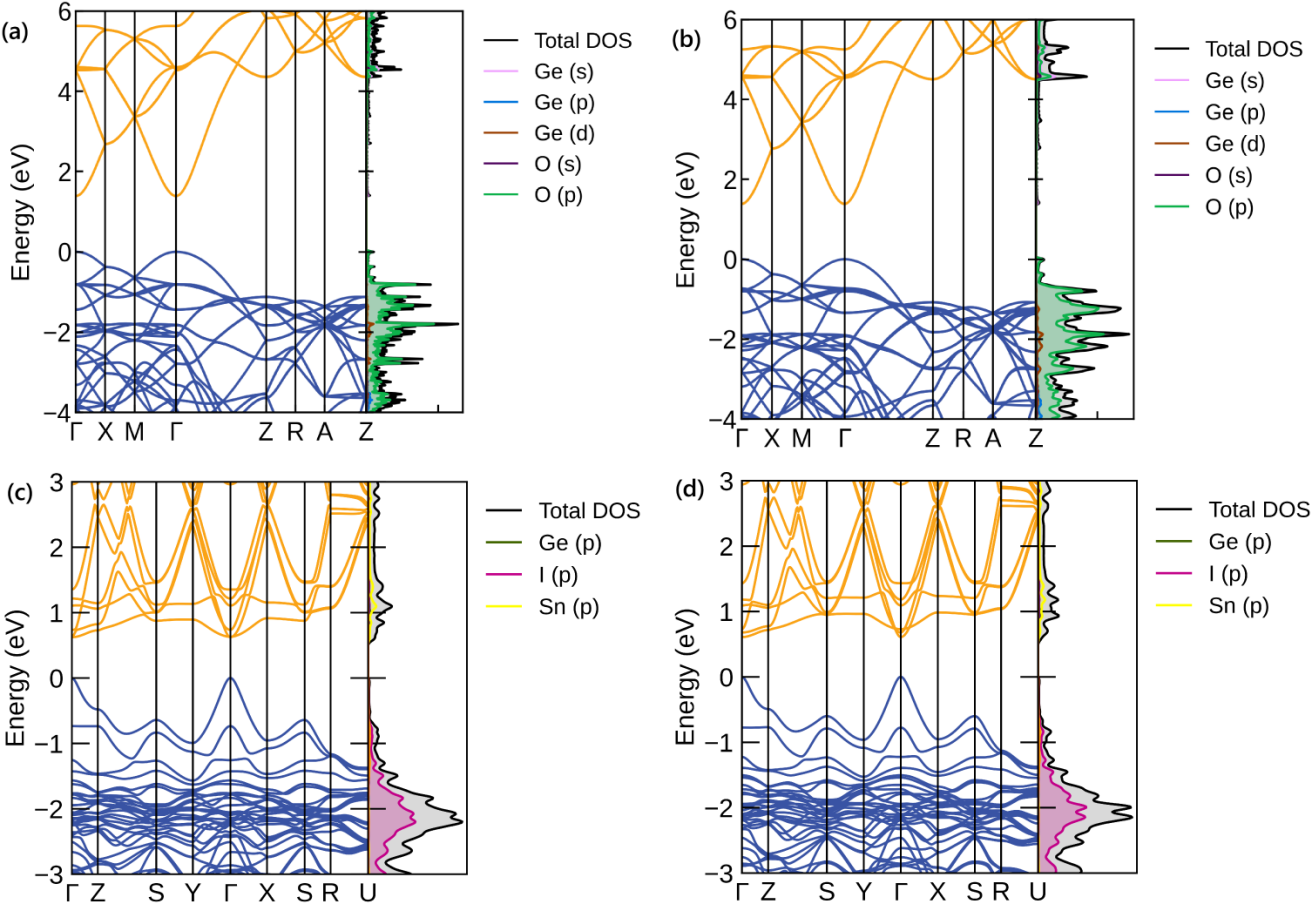
PAW/PBE-calculated band structure and DOS for (a) relaxed and (b) stressed (2 × 2) supercell of *r*-GeO_2_. (c) and (d) are the same for the γ-Cs­(Sn_1-x_,Ge_x_)­I_3_ (*x*=0.25) unit cell.

**Table 2:** PAW/PBE Calculated Bandgap and Workfunction (Wf) for the Perovskite Slabs Here Considered^a^

	CsSnI_3_	Cs(Sn_1–*x*_Ge_*x*_)I_3_ (*x* = 0.25)				
	SnI_2_-Term (Rel)	MI_2_-Term (M_1_=Sn,Ge) (Rel)	MI_2_-Term (M_1_=Sn,Ge) (Interf, **3**)	MI_2_-Term (M_2_=Ge) (Interf, **4**)	CsI-Term (M_1_=Sn,Ge) (Interf, **1**)	CsI-Term (M_2_=Ge) (Interf, **2**)
*E*_gap_ (eV)	0.86	0.81	0.73	0.83	0.74	0.54
Wf (Φ, eV)	5.02 (4.85)^80^	5.12	5.33	5.51	3.71	3.76

a “*rel*” refers to relaxed, no constraints systems; “*interf*” refers to values calculated keeping fixed the lateral parameters to those of the final interface. For CsI-terminated systems “M” refers to the first metal layer just immediately beneath the surface. In parenthesis is reported the experimental value for CsSnI_3_.

**Figure 6:**
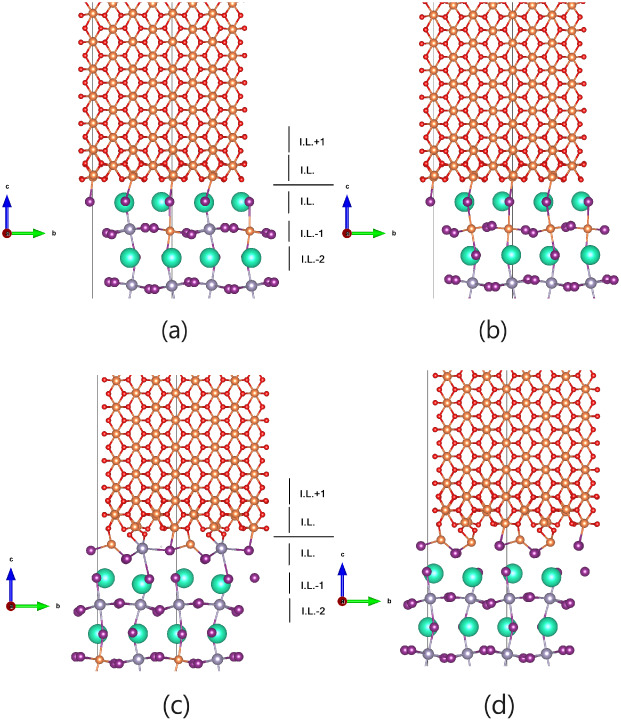
PAW/PBE-optimized structure of the four considered interfaces: CsI-terminated with (a) (Sn,Ge)-mixed layer beneath the perovskite surface layer (M_1_, **1** in Table 3) (b) Ge only (M_2_) (**2**); MI_2_-terminated with (c) (Sn,Ge)-mixed termination (M_1_) at the perovskite surface (**3**), (d) Ge only at the perovskite surface (M_2_, **4**). For the sake of visualization a (2 × 2) supercell is reported. [Mauve: Sn; Orange: Ge; Purple: I; Red: O; Cyan: Cs atoms]. Figures (a) and (c) illustrate the layer-resolved breakdown of the interfaces used for PDOS analysis, projected onto individual layers (see Supplementary Figure S4).

Before we tackle the study of electronic properties, we focus on the structural analysis of the interfaces considered here. Let us begin with the CsI-terminated system with M = Sn, Ge (M_1_) in the layer immediately below the surface (shown in Figure 6a). The characteristic structure of Ge halide perovskites is generated by three short bonds (2.83, 2.83, and 2.86 Å) and three long bonds (3.18, 3.28, and 3.32 Å), giving rise to the umbrella-like shape typical of such compounds,^34^ as we previously discussed. When only germanium is present in the layer beneath the surface (M_2_=Ge only, Figure 6b), we observe the same phenomenon reported. In this case, also, there are three long bonds (3.03, 3.41, and 3.57 Å) and three short ones (2.73, 2.82, and 2.95 Å). It is interesting to note that of the three long bonds, one is at the surface (3.03 Å), which causes a partial separation of the oxide from the perovskite. In fact, the germanium in the oxide layer forms a Ge–I bond at the interface of 2.84 Å, which is significantly shorter than the apical Ge–I bond discussed earlier: the iodine atoms at the surface tend to migrate from the perovskite, therefore passivating the oxide component.

Structurally, MI_2_-terminated systems are of special interest. In detail, starting from the mixed composition (M_1_=Sn,Ge), we observe how the coexistence of these two elements at the perovskite surface results in a particularly heterogeneous interface geometry (shown in Figure 6c). As in previous cases, Ge atoms at the terminating layer of the perovskite form three short bonds and three longer ones. With regard to the former, two bonds are formed between Ge and O (2.03 and 2.23 Å), while the third is formed between Ge and I (2.76 Å), the latter being a remnant of the initial perovskite structure. For the Sn atom, it forms up to six bonds, four with iodine (ranging from 2.48 to 3.52 Å) and two shorter ones with oxygen (2.17 and 2.25 Å, respectively), revealing the more marked ionic nature for such interface, as also witnessed by the two- and 3-fold coordinated I atoms at the interface.

The last investigated structure (reported in Figure 6 d) presents germanium atoms only at the surface of the perovskite (M_2_ = Ge). The optimized structure reveals how the surface perovskite layer, here consisting of GeI_2_, is completely detached from its pristine position and torn away by the oxide. In this configuration, the oxide saturates its surface layer, leaving the perovskite terminated with the subsuperficial SnI_2_ layer.

A way to evaluate the stress at the interface is to consider the total stress energy defined as the difference between the total energies of the interface systems and the sum of the energies of the corresponding bulk GeO_2_ and Cs­(Sn_1__–*x*_Ge_*x*_)­I_3_ phases. Although this approach is certainly valuable as it allows the calculation of both chemical and mechanical stress at the interface, it is probably more suitable and quantitative for periodic double-interface models,^81^ where surface effects are minimized and, more importantly, no layers are kept frozen. In the present case, we evaluate the energetics of the system by calculating the adhesion energy, i.e., the opposite of the energy required to separate the surfaces that form the interface, as

2 $$E_{adh}=E_{interf}-\left(E_{Cs(Sn,Ge)I_{3}‐slab}+E_{GeO_{2}‐slab}\right)$$

where *E*_interf_ is the energy of the optimized interface, and $E_{Cs(Sn,Ge)I_{3}‐slab}$ and $E_{GeO_{2}‐slab}$ are the energies of the slab of Cs­(Sn_1–*x*_Ge_*x*_)­I_3_ and of GeO_2_, respectively. The results are reported in Table 3 and reveal that the MI_2_-terminated slab tends to form more stabilized interfaces in both the case of pure Ge on the surface and in the mixed case.

**Table 3:** PAW/PBE Calculated Adhesion Energy and Bandgap of the Four Interfaces Here Investigated

Interface (Termination)	Adhesion Energy (eV, J m^–2^)	Bandgap (eV)
**1** (CsI–, M_1_=Sn,Ge)	–2.51 (−0.529)	0.49
**2** (CsI–, M_2_=Ge only)	–2.47 (−0.521)	0.39
**3** (MI_2_–, M_1_=Sn,Ge))	–4.40 (−0.928)	(*metallic*)
**4** (MI_2_–, M_2_=Ge only))	–4.67 (−0.985)	(*metallic*)

For all the interfaces we have then calculated the electronic properties, i.e., DOS and valence band offset (VBO). Given the particularly large dimensions of our interface models, their electronic properties were evaluated only at the DFT level. It is well known that standard DFT functionals systematically underestimate excited-state properties, such as band gaps, due to their mean-field, ground-state nature. However, this limitation does not compromise the present analysis, as the underestimation is largely systematic and comparable across all the investigated systems. Consequently, relative trends and comparisons remain meaningful. For the calculation of VBO we have employed the formalism of van de Walle and Martin,^82^ successfully applied to several other interfaces:^74^

3 $$\Delta \epsilon_{VBO}=\left(\epsilon_{V}^{\mathrm{Cs}({\mathrm{Sn}}_{1-x}{\mathrm{Ge}}_{x})I_{3}}-{\mathrm{V̅}}_{\mathrm{bulk}}^{\mathrm{Cs}({\mathrm{Sn}}_{1-x}{\mathrm{Ge}}_{x})I_{3}}\right)-\left(\epsilon_{V}^{{\mathrm{GeO}}_{2}}-{\mathrm{V̅}}_{bulk}^{{\mathrm{GeO}}_{2}}\right)+\Delta \mathrm{V̿}$$

where $\epsilon_{V}^{\mathrm{Cs}({\mathrm{Sn}}_{1-x}{\mathrm{Ge}}_{x})I_{3}}$ and $\epsilon_{V}^{{\mathrm{GeO}}_{2}}$ are the valence band maxima of Cs­(Sn_1–*x*_Ge_*x*_)­I_3_ and GeO_2_, respectively, while ${\mathrm{V̅}}_{\mathrm{bulk}}^{\mathrm{Cs}({\mathrm{Sn}}_{1-x}{\mathrm{Ge}}_{x})I_{3}}$ and ${\mathrm{V̅}}_{bulk}^{{\mathrm{GeO}}_{2}}$ are the nanosmoothed averaged electrostatic potentials in Cs­(Sn_1–*x*_Ge_*x*_)­I_3_ and GeO_2_ slabs, respectively. Δ*V̿* is the difference between the nanosmoothed averaged electrostatic potentials at the interface. We calculated a Δ*ϵ*_VBO_ for the four interfaces, which ranges between 0.39 eV (interface **1**) and 1.69 eV (interface **4**), revealing the completely different nature of the interfaces as a function of the perovskite termination. The planar averaged electrostatic potential for the four interfaces here investigated is reported in Figure S2 in Supporting Information. We additionally show the DOS for the four interfaces in Figure 7, along with the Projected Density of States onto the different layers at the interfaces (see Figure S4 in Supporting Information).

**Figure 7:**
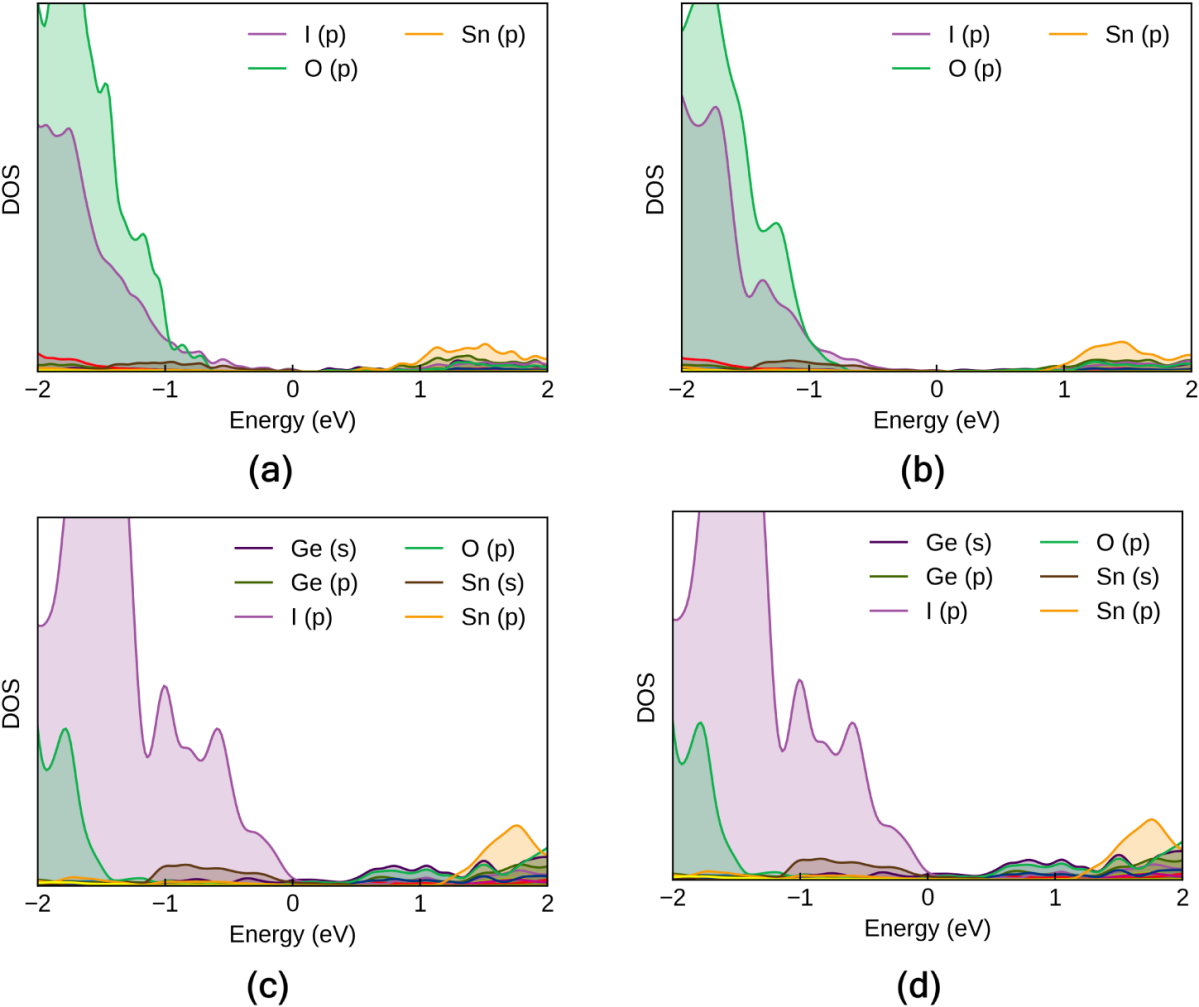
Total and Projected Density of States for the four interfaces formed by crystalline *r*-GeO_2_ and Cs­(Sn_1–*x*_Ge_*x*_)­I_3_ (*x* = 0.25). CsI-terminated with (a) mixed (Sn, Ge) layer beneath the perovskite surface (M_1_, **1** in Table 3) (b) Ge only in the layer beneath the perovskite surface (M_2_, **2**); MI_2_-terminated with (c) mixed (Sn, Ge) at the perovskite surface (M_1_, **3**), (d) Ge only at the perovskite surface (M_2_, **4**).

While the two CsI-terminated interfaces are characterized by a bandgap, both MI_2_-terminated interfaces are metallic. This finding is consistent with previously reported data—mostly ascribed to strain-induced effects—for interfaces between silicon and methylammonium lead iodide (MAPbI_3_), where analogous termination-dependent behavior was observed: PbI_2_-terminated interfaces are metallic, whereas the MAI-terminated ones are characterized by a bandgap.^74^ To gain further insight, we analyzed the squared modulus of the band-edge wave functions for the CsI-terminated interfaces The first investigated interface (**1**) has a VBM mainly constituted by I 5*p* orbitals (61%), by Sn 5*s* orbitals (34.5%), and by Ge 4*s* ones (∼4.0%), the latter belonging to the perovskite slab. The CBM is at variance constituted by 61% of Ge 4*s* orbitals and ∼28% of O 2*p*, these two latter belonging to the GeO_2_ slab and mostly localized at the interface. Similar behavior is observed for the second interface (**2**) where the VBM is located mainly in the inner region of the perovskite and the CBM in the GeO_2_ layer forming the interface. The overall picture that emerges from this orbital (de)­localization is that the interface region acts as a recombination center, due to the extremely localized nature of the electrons at the interface.

To complete the picture, we establish a parallelism between crystalline and amorphous germania and their influence on the electronic features of the interfaces under investigation. We used the same perovskite slabs, onto which we placed the amorphous germania constructed following the procedure described in the Computational Details section. As a result, we obtain three stable interfaces (those formed with MI_2_-terminated (M_1_ = Sn, Ge) are thermodynamically unstable), whose structures are reported in Figure 8 and whose adhesion energies are reported in Table 4. The values—clearly more exothermic than those of the crystal–crystal counterparts—reflect the enhanced formability of the amorphous-crystalline interfaces because of the disordered amorphous structure and the lack of a long-range order. These results similarly highlight the larger tolerance and flexibility of the amorphous phase when interacting with the more-ordered atomic framework of the crystalline perovskite slab.

**Figure 8:**
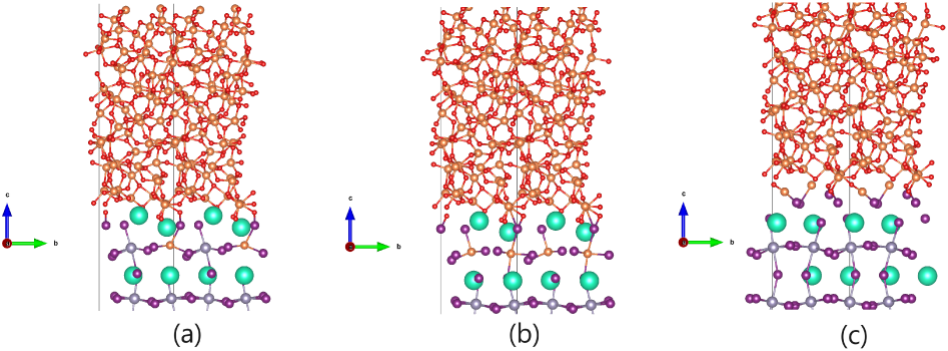
PAW/PBE-optimized structure of the four interfaces formed by amorphous germania here considered: CsI-terminated with (a) mixed (Sn, Ge) layer beneath the perovskite surface; (b) Ge only layer beneath the perovskite surface; (c) MI_2_-terminated with Ge only at the perovskite surface. For the sake of visualization a (2 × 2) supercell is reported. [Mauve: Sn; Orange: Ge; Purple: I; Red: O; Cyan: Cs atoms].

**Table 4:** PAW/PBE Calculated Adhesion Energy and Bandgap of the Investigated Three Stable Interfaces with Amorphous GeO_2_

Interface (Termination)	Adhesion Energy (eV, J m^–2^)	Bandgap (eV)
**1a** (CsI–, M_1_=Sn,Ge)	–5.76 (−1.214)	(*metallic*)
**2a** (CsI–, M_2_=Ge only)	–6.86 (−1.446)	0.013
**4a** (MI_2_–, M_2_=Ge only)	–7.74 (−1.631)	0.007

The modulus of the VBM and CBM wave functions provides a direct, microscopic visualization of the real-space localization of the frontier orbitals at the interface, which is a widely accepted indicator of charge transfer propensity and chemical reactivity in DFT studies of heterostructures.^74,83,84^ The most relevant outcome of our analysis is the completely different behavior of the band edge states at the interfaces involving amorphous versus crystalline GeO_2_. For the latter (as shown in Figure 9), we observed that the interfaces act as recombination centers, while the amorphous germania interfaced with the (Sn, Ge)-alloyed perovskite shows an opposite trend. The VBM is indeed localized in the germania, corresponding to an enhanced localization of electrons in such a region, as shown in Figure 10. This means that amorphous oxide tends to preserve the perovskite layer and prevent its eventual oxidation, a result that supports previous experimental works^40^ that clearly claim the beneficial effect in terms of stability of an amorphous oxide layer on top of mixed-halide (Sn,Ge) perovskites. Before concluding, we should spend a few words of caution regarding our findings for both crystalline and amorphous oxide systems. While we observe that adhesion energies clearly favor MI_2_-terminated Cs­(Sn,Ge)­I_3_/GeO_2_ interfaces, the resulting metallic character (small/no-gap states) and large VBO (in the crystalline case) arise from static DFT-PBE models of reactive crystal/crystal contacts, where optimization induces GeI_2_-like species akin to PbI_2_ formation under atomic layer-deposited SnO_2_ ^85^ and dangling bonds from rigid MI_2_ terminations.^74^ Amorphous GeO_2_ cases, modeled following the experimentally reported procedure,^40^ retain residual strain from lattice averaging, likely exaggerating gap states versus dynamic deposition. Prepassivation of the oxide electron transport layer^86^ would definitely mitigate recombination risks and band misalignment in devices. Not secondarily, the level of theory we employed is by construction unable to capture the exact nature of the bandgap (mostly for what concerns the excited-state properties). On the other hand, more accurate methods such as HSE06 hybrids or GW are computationally prohibitive for our large supercells due to their relevant scaling with system size.

**Figure 9:**
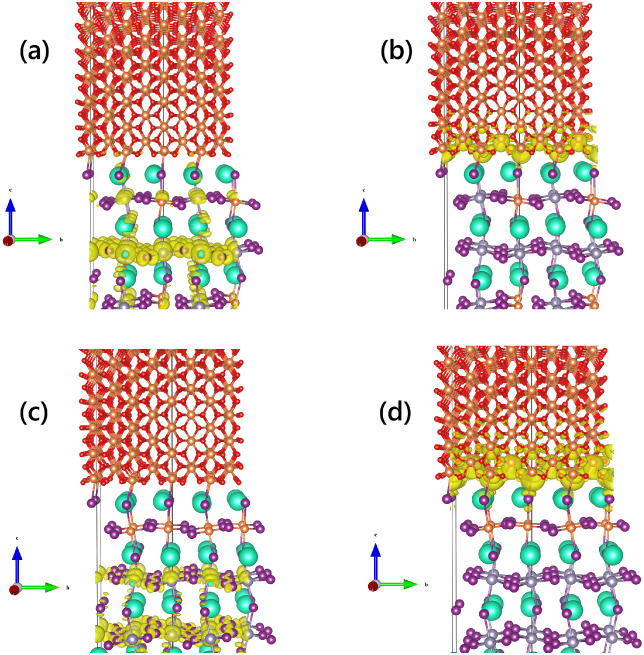
(a) Valence Band Maximum and (b) Conduction Band Maximum wave function square modulus for the **1** interface. (c) and (d) are the same for the **2** interface. For the sake of visualization a (2 × 2) supercell is reported. [Mauve: Sn; Orange: Ge; Purple: I; Red: O; Cyan: Cs atoms] (isosurface level 3 × 10^–4^ *e*/Bohr^–3^).

**Figure 10:**
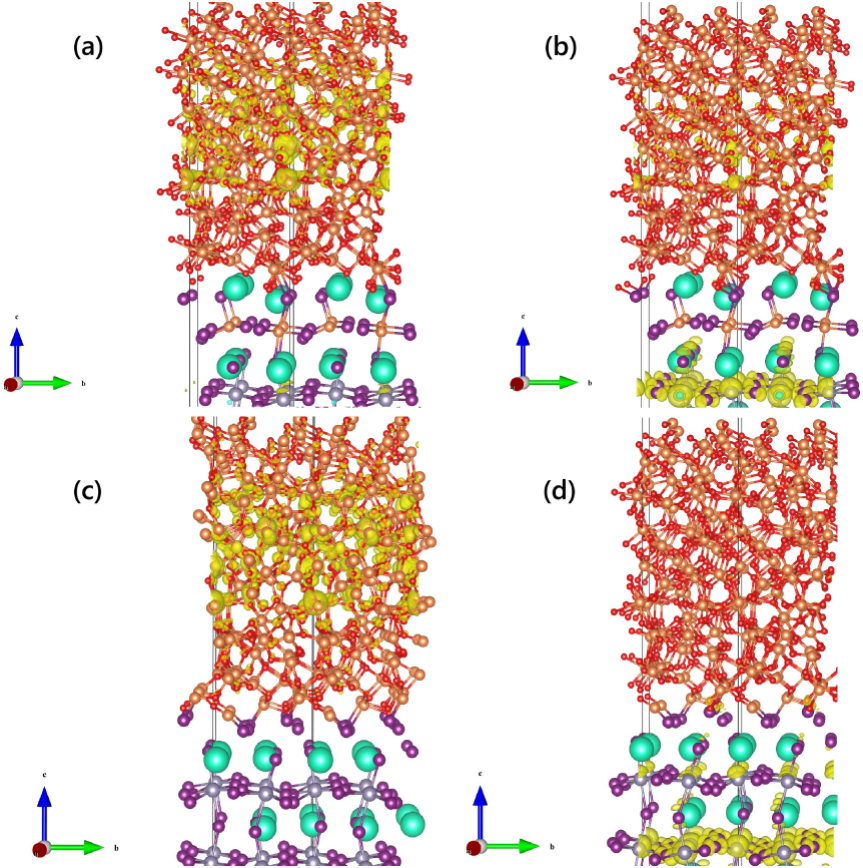
(a) Valence Band Maximum and (b) Conduction Band Maximum Wave function square modulus for the **2a** interface. (c) and (d) are the same for the **4a** interface. For the sake of visualization a (2 × 2) supercell is reported. [Mauve: Sn; Orange: Ge; Purple: I; Red: O; Cyan: Cs atoms] (isosurface level 3 × 10^–4^ *e*/Bohr^–3^).

## Conclusions

Given the technological relevance of lead-free halide perovskites, we have investigated interfaces between oxides and lead-free (Sn,Ge)-mixed halide perovskites. We have at first considered the three perovskite parental compounds, i.e., CsSnI_3_ and CsGeI_3_, and their alloyed system Cs­(Sn_1–*x*_Ge_*x*_)­I_3_. The comparison of their optical spectra demonstrates that pronounced excitonic red shifts and intensity enhancements are a distinctive feature of CsSnI_3_ only, whereas excitonic effects remain weak in the Ge-based and mixed systems, whose responses closely follow the independent-particle behavior. Markedly different behavior is observed studying amorphous and crystalline germania when interfaced with Cs­(Sn_1–_ *_x_* Ge_*x*_)­I_3_. We both considered MI_2_- (M_1_=Sn,Ge; M_2_=Ge only) and CsI-(M_1_=Sn,Ge; M_2_=Ge only at the layer immediately under the CsI outmost layer) terminated perovskites and observed that the former form more stable interfaces (more exothermic adhesion energy) with *r*-GeO_2_ compared to the latter ones. Both MI_2_-terminated perovskite crystalline interfaces show a metallic character, while both considered CsI-terminated perovskite interfaces maintain a gap.

For interfaces with crystalline rutile GeO_2_, the charge transfer is from the valence band maximum localized in the Cs­(Sn_1–_ *_x_* Ge_*x*_)­I_3_ perovskite, to the conduction band minimum, which is localized in the oxide part at the very interface. When the surface of the perovskite is terminated with GeI_2_, such termination tends to drift toward GeO_2_, providing an interesting structural characteristic common in both crystalline and amorphous systems (iodine passivation of the GeO_2_ layer). For interfaces formed with amorphous GeO_2_, the electronic characteristics are more heterogeneous compared to the case of crystalline rutile GeO_2_: the interfaces with a bandgap are indeed those formed either with CsI- or GeI_2_-terminated perovskite. For interfaces with amorphous GeO_2_, charge transfer clearly takes place between the amorphous germania inner region (valence band maximum) and the perovskite inner region (conduction band minimum). This latter result is in fair agreement with experiments that reveal the benign formation of an oxide amorphous layer on top of the (Sn, Ge)-mixed perovskite. Such a layer is found to protect the perovskite slab and to prevent its oxidation, as clearly confirmed from our calculations.

## Supplementary Material



## Notes

PBE + SOC-calculated band structures for bulk perovskite systems; electrostatic potential profiles across the interfaces between the perovskite and crystalline *r*-GeO_2_; surface formation energies and workfunctions of the perovskite terminations; density of states (DOS) of Ge atoms in the perovskite slabs closest to the interface formed with *r*-GeO_2_; layer-by-layer contribution to the projected density of states (PDOS) at the perovskite/crystalline *r*-GeO_2_ interface (PDF)
