# Supplementary material for: Protective Role of Oxides on Pb-Free Halide Perovskite Surfaces: Interfacial Effects and Excitonic Optical Properties from First-Principles
Source: ACS Appl Mater Interfaces. 2026 Jun 8;18(23):32782–94. doi: 10.1021/acsami.6c00873 (PMC13288393; doi:10.1021/acsami.6c00873)
Supplement: Supplementary file 1 [file am6c00873_si_001.pdf]

Supporting Information:

Protective Role of Oxides on Pb-free Halide Perovskite Surfaces: Interfacial Effects and Excitonic Optical Properties from First-Principles

Maurizia Palummo*,[†,‡] Costanza Borghesi,[‡,¶,§] Azusa Muraoka,[∥] Suraya Shaban,[⊥] Shuzi Hayase,[⊥] Daniele Varsano,[‡] Koichi Yamashita,[#] and Giacomo Giorgi*,[¶,§,‡,@]

†*Department of Physics & INFN, Universitá di Roma "Tor Vergata," Via della Ricerca Scientifica 1, 00133 Roma, Italy.*

‡*Centro S3, CNR-Istituto Nanoscienze, Via G. Campi 213/a, Modena, 41125, Italy, Italy.*

¶*Department of Civil & Environmental Engineering (DICA), The University of Perugia, Via G. Duranti 93, 06125 Perugia, Italy.*

§*CIRIAF - Interuniversity Research Centre, University of Perugia, Via G. Duranti 93, 06125 Perugia, Italy.*

∥*Graduate School of Science, Japan Women's University, Tokyo 112-8681, Japan.*

⊥*i-PERC, The University of Electro-Communications, 1-5-1 Chofugaoka, Chofu, Tokyo 182-8585, Japan.*

#*Graduate School of Nanobioscience, Yokohama City University, Yokohama, 236-0027, Japan.*

@*CNR-SCITEC, 06123 Perugia, Italy.*

E-mail: maurizia.palummo@roma2.infn.it;giacomo.giorgi@unipg.it

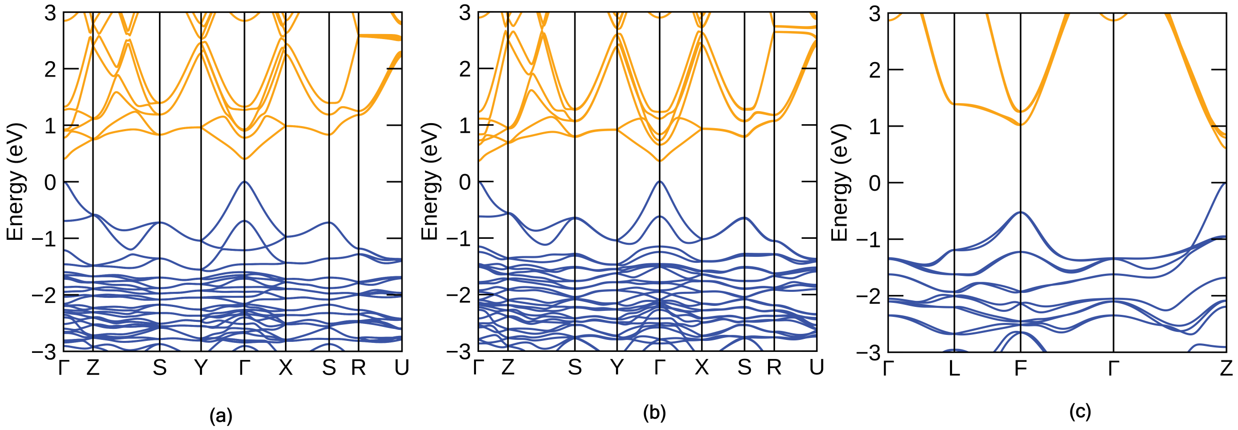

Figure S1: PBE+SOC calculated bandstructure for (a) γ-$CsSnI_3$, (b) $Cs(Sn_{1-x}Ge_x)I_3$ (x=0.50) alloyed system (c) $CsGeI_3$

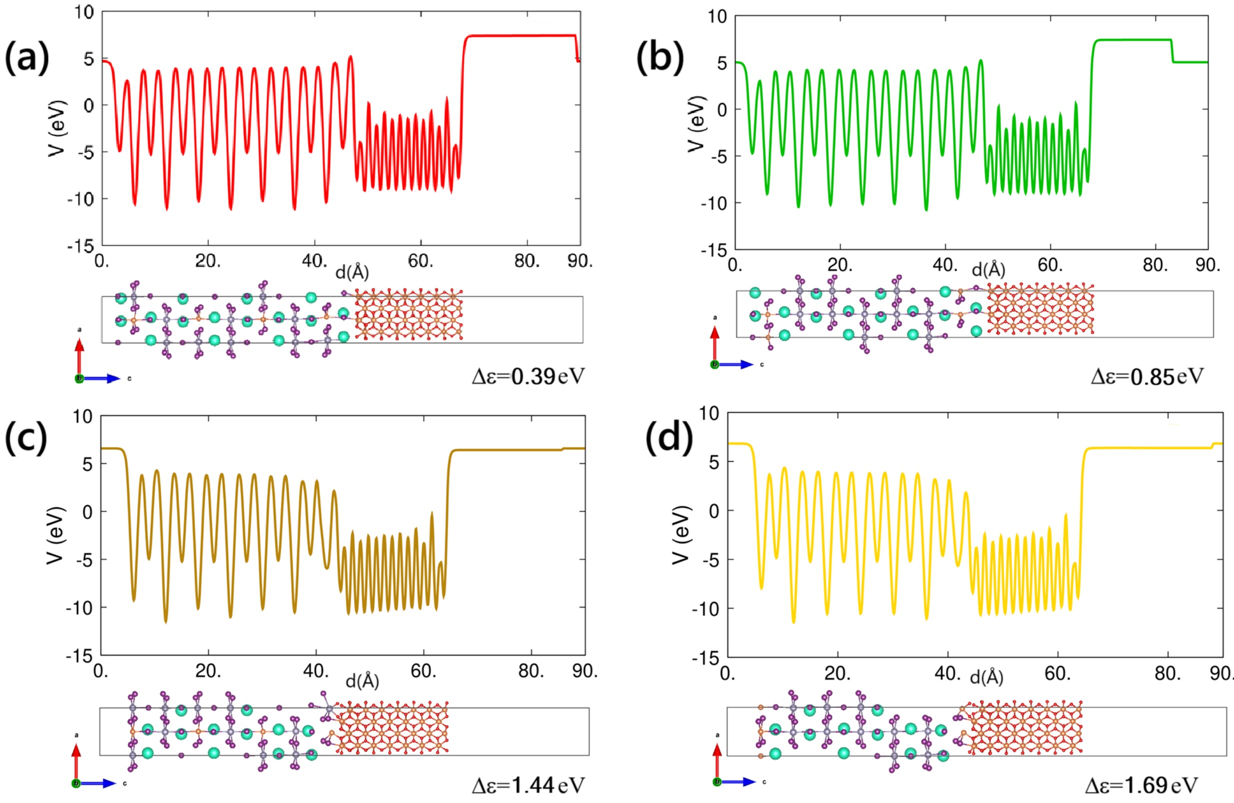

Figure S2: Electrostatic potential profiles for the four investigated $Cs(Sn_{1-x}Ge_x)I_3/r{-}GeO_2$ (x=0.25) interfaces along with the PAW/PBE calculated Valence Band Offset (eV): (a,b) CsI- and (c,d) MI_2-terminated perovskites.

Table 1: Surface Formation Energy of the different terminations considered and calculated according to Eq.1. Workfunctions (eV) for the same surfaces are reported. Lateral parameters are fixed to those optimized for $Cs(Sn_{1-x}Ge_x)I_3$ (x=0.25)

	CsI-term (**1**) (M=Sn, Ge)	CsI-term (**2**) (M=Ge)	MI_2-term (**3**) (M=Sn, Ge)	MI_2-term (**4**) (M=Ge)
SFE (eV)	0.113	0.104	0.285	0.246
Φ (eV)	3.60	3.51	5.20	5.23

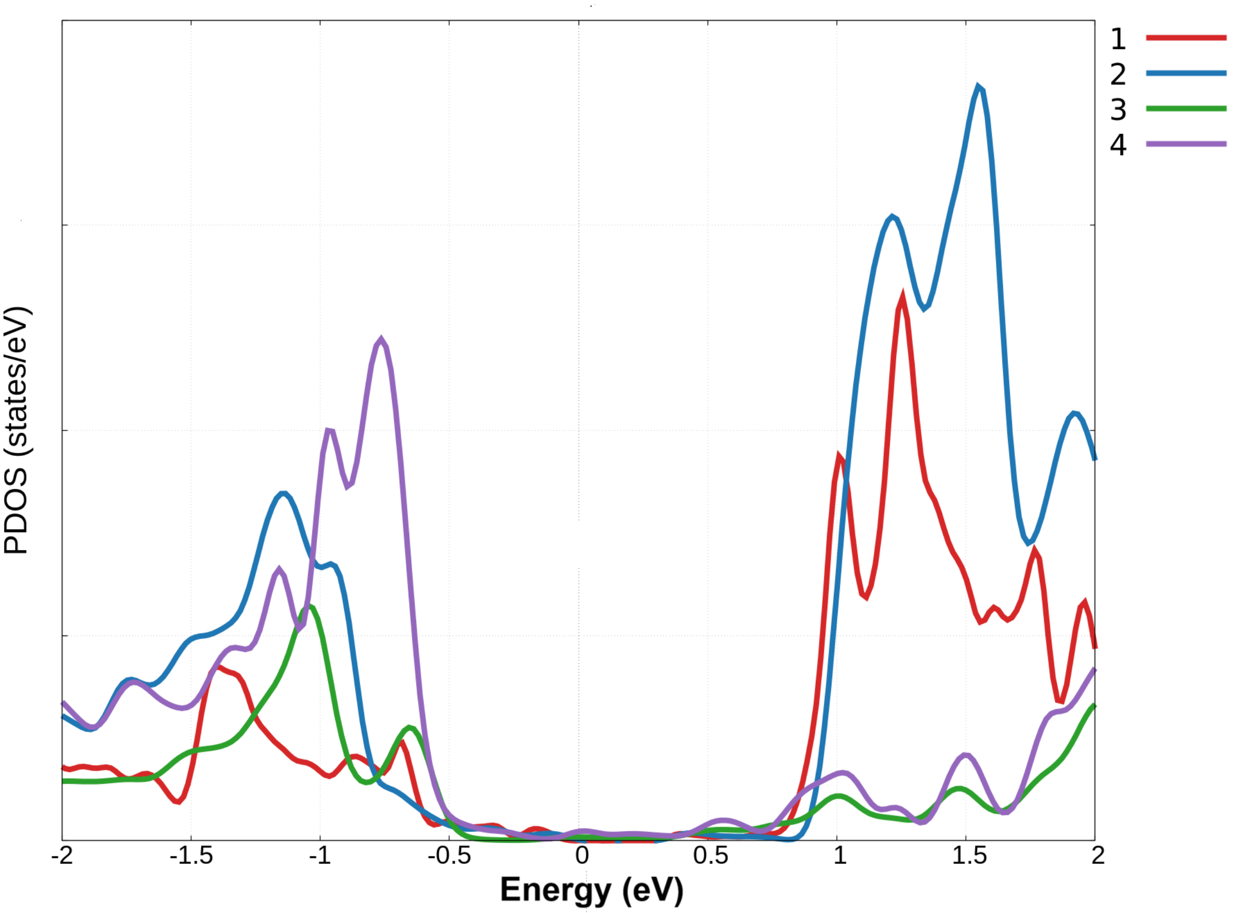

Figure S3: DOS of the Ge atoms at the surfaces in the perovskite slab. For the sake of visualization, the DOS have been significantly enlarged.**1-4** refer to the different interfaces considered in the the present work (and as listed in Table 3 in the main text).

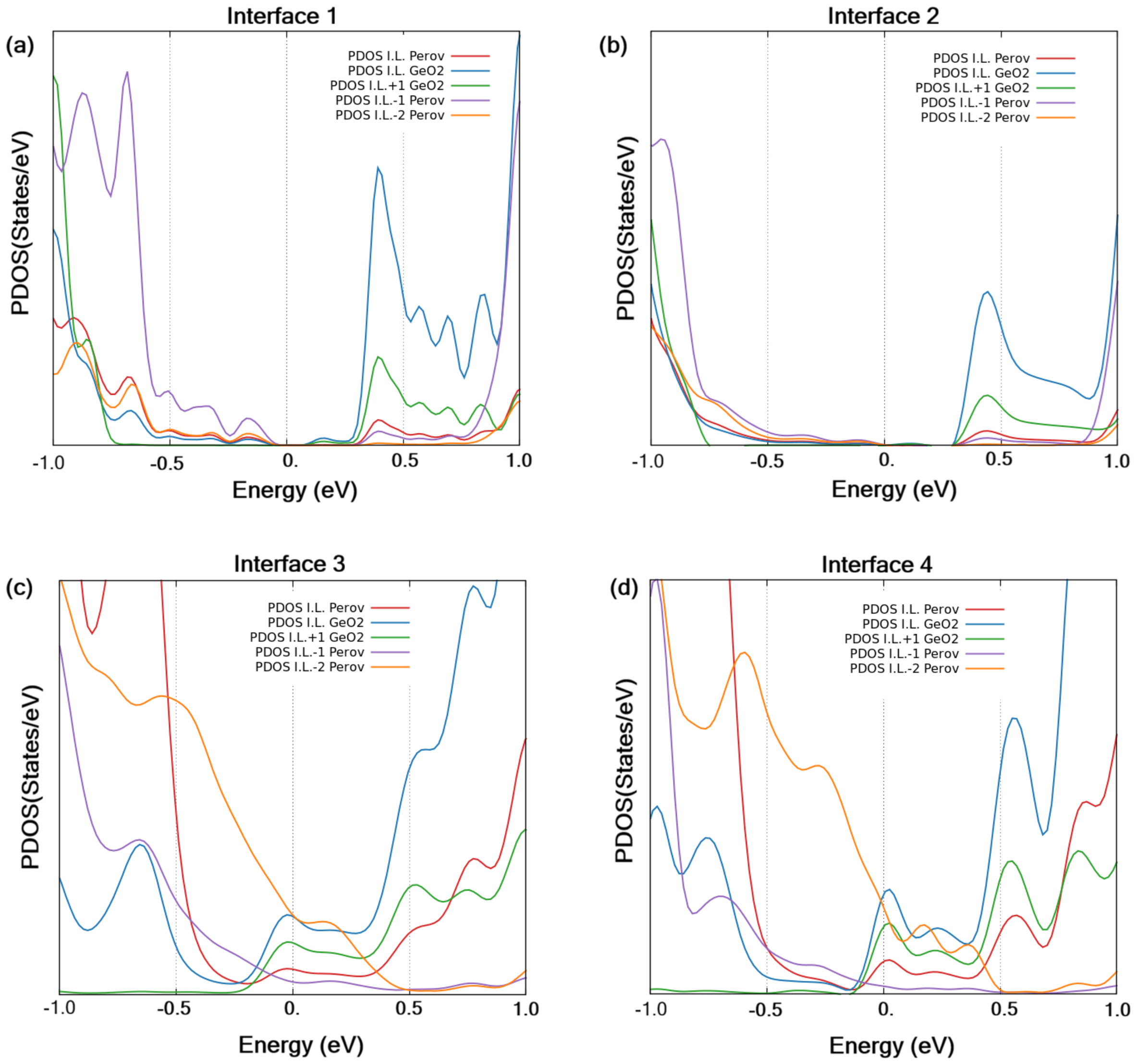

Figure S4: PDOS of the layers contributing to the interface. CsI-terminated with (a) mixed (Ge,Sn) at perovskite top layer (interface **1** in Table 3 in the main text) (b) Ge only at the top layer (interface **2**); MI_2-terminated with (c) mixed (Ge,Sn) at perovskite surface (interface **3**), (d) Ge only at the top layer (interface **4**). For the layer-by-layer contribution interpretation/formalism, see Figure 6 in the main text.
